# Supplementary material for: Heat adaptation of phage T7 under an extended genetic code
Source: Virus Evol. 2021 Dec 1;7(2):veab100. doi: 10.1093/ve/veab100 (PMC8923235; doi:10.1093/ve/veab100)
Supplement: veab100_Supp [file veab100_supp.zip › Sequencing_Analysis.zip!/Sequencing_Analysis/S1/evidence/JC_SIDE_2_57.html]

BRESEQ :: Evidence


| New junction evidence | | | | | | | | | | | |
| --- | --- | --- | --- | --- | --- | --- | --- | --- | --- | --- | --- |
|  | | seq id | position | reads (cov) | reads (cov) | score | skew | freq | annotation | gene | product |
| \* | ? | NC\_001604.1 | = 13586 | 3 (0.000) | 50 (0.010) | 23/592 | 37.3 | 30.3% | intergenic (–/–) | *–/–* | –/– |
|  | ? | NC\_001604.1 | 14356 = | 227 (0.050) |  |  |  |  | intergenic (–/–) | *–/–* | –/– |
| Rejected: Coverage evenness skew score above cutoff. | | | | | | | | | | | |

Only 100 of 4642 total aligned reads displayed.  

`GACAGACTGGCTGGAAGAAACCAGAAGTTACCAAGCTGAGTCATAAGGTGTTCGATAAGGACACTATGACCCACATCAAGGCTGGTGATTGGGTTAAGGTTGACATGGGAGTTGTTGGTGGATACGGCTACGTCCGCTCAGTTAGTGGCAAATATGCACAAGTGTCATACATCACAGGTGTTACTCCACGCGGTGCAATCGTTGCCGATAAGACCAACATGATTCACACAGGTTTCTTGACAGTTGTTTCATATGAAGAGATTGTTAAGTCACGATAATCAATAGGAGAAATCAATATGATCGTTTCTGACATCGAAGCTAACGCCCTCTTAGAGAGCGTCACTAAGTTCCACTGCGGGGTTATCTACGACTACTCCACCGCTGAGTACGTAAGCTACCGTCCGAGTGACTTCGGTGCGTATCTGGATGCGCTGGAAGCCGAGGTTGCACGAGGCGGTCTTATTGTGTTCCACAACGGTCACAAGTATGACGTTCCTGCATTGACCAAACTGGCAAAGTTGCAATTGAACCGAGAGTTCCACCTTCCTCGTGAGAACTGTATTGACACCCTTGTGTTGTCACGTTTGATTCATTCCAACC  >  NC_001604.1/14057‑14656`  

                                                                                                                                                                                                                                                                                                           `|`                                                                                                                                                                                                                                                                                                              
`agacgactacgagaagtttcgatggagagtcccattctaatacgactcactaaatgagacacaccatgttcaaactgattaagaagttaggccaactgctggttcgtatgtacaacgtggaggccaagcgactgaacgatgaggctcgtaaagaggccacacagtcacgcgctctggcgattcgctccaacgaactggctgacagtgcatccactaaagttaccgaggctgcccgtgtggcaaaccaagctcaacagctttccaaattctttgagtaatcaaTAGGAGAAATCAATATGAt                                                                                                                                                                                                                                                                                                             <  1:407058‑M2/19‑1 (MQ=255)`  
`agacgactacgagaagtttcgatggagagtcccattataaaacgacacaagaaaggagacacaccatgttcaaactgattaagaagttaggccaactgctggttagtatgtacaacgtggaagccaagcgactgaacgatgaggctcgtaaagaggccacacagtcacgcgctctggcgattcgctccatcgaactggctgacagtgcatccactaaagttaccgaggctgcccgtgtggcaaaccaagcgcaacagctttccaaattctttgagtaatcaaTAGGAGAAATCAATATGAt                                                                                                                                                                                                                                                                                                             <  1:425774‑M2/19‑1 (MQ=255)`  
     `ctacgagaagtttcgctggagagtcccattctaatacgactcactaaaggagacacaccatgttcaaactgattaagaagttaggccaactgctggttcgtatgtacaacgtggaagccaagcgactgaacgatgaggctcgtaaagaggccacacagtcacgcgctctggcgattcgctccaacgaactggctgacagtgcatccactaaagttaccgaggctgcccgtgtggcaaaccaagctcaacagctttccaaattctttgagtaatcaaaCAGGAGAAATCAATATGATCGttt                                                                                                                                                                                                                                                                                                        >  1:100160‑M2/278‑301 (MQ=255)`  
                 `ttcgctggagcgttccattctaatacgactcactaaaggagacacaccatgttcaaactgattaagaagttaggccaactgctggttcgtatgtacaacgtggacgccacgcgactgaacgatgaggctcgtcaagaggccacacagtcacgcgctctggcgattcgctccaacgaactggctgacagtgcatccactaaagttaccgaggctgcccgtgtggcaaaccaagctcaacagctttcccaattctttgagtaatcaaTAGGAGAAACCAATATGAAcgtttctgaactgtctc                                                                                                                                                                                                                                                                                            >  1:448438‑M2/266‑293 (MQ=255)`  
                  `cgctggagagtcccattctaatacgactcactaaaggagacacaccatgttcaaactgattaagaagttaggccaactgctggttcgtatgtacaacgtggaagccaagcgactgaacgatgaggctcgtaaagaggccacacagtcacgcgctctggcgattcgctccaacgaactggctgacagtgcatccactaaagttaccgaggctgcccgtgtggcaaaccaagctcaacagctttccaaattctttgagtaatcaaaCAGGAGAAATCAATATGATCGTTTCTGACATCGAAGc                                                                                                                                                                                                                                                                                           >  1:65375‑M2/265‑301 (MQ=255)`  
                           `gtcccattctaatacgactcactaaaggagacacaccatgttcaaactgattaagaagttaggccaactgctggttcgtatgtacaacgtggaagccaagcgactgaacgatgaggctcgtaaagaggccacacagtcacgcgctctggcgattcgctccaacgaactggctgacagtgcatccactaaagttaccgaggctgcccgtgtggcaaaccaagctcaacagctttccaaattctttgagtaatcaaaCAGGAGAAATCAATATGATCGTTTCTGACATCGAAGCTAACgcccc                                                                                                                                                                                                                                                                                  >  1:214075‑M2/256‑300 (MQ=255)`  
                              `ccattctaatacgacgcacgaaaggagacacaccatgttcaaactgatgaagaagttaggccaactgctggttcgtatgtacaacgtggaagccaagcgactgaacgatgaggctcgtaaagaggccacacagtcacgcgctctggcgattcgctccaacgaactggctgacagtgcatccactaaagttaccgaggctgcccgtgtggcaaaccaagctcaacagctttccaaattctttgagtaatcaaaCAGGAGAAATCAATATGATCGTTTCTGACATCGAAGCTAACGCCCTCtt                                                                                                                                                                                                                                                                               <  1:68624‑M2/49‑1 (MQ=255)`  
                                `cattctaatacgactcactaaaggagacacaccatgttcaaactgattaagaagttaggccaactgctggttcgtatgtacaacgtggaagccaagcgactgaacgatgaggctcgtaaagaggccacacagtcacgcgctctggcgattctctccaacgaactggctgacagtgcatccactaaagttaccgaggctgcccgtgtggccaaccaagctcaacagcattccaaattctttgagtaatcaaTAGGAGAAATCAATATGATCGTTTCTGACATCGAAGCTAACGCCCTCTTag                                                                                                                                                                                                                                                                             >  1:148656‑M2/251‑301 (MQ=255)`  
                                `attctaatacgactcactaaaggagacacaccatgttcaaactgattaagctgttaggccaccttctggttcgtatgtacaacttggaagtcaagcgcttaaacgatgagcctcgtaaagtggcctcacagtcacgcgttctggcgattcgctacaacgaactagctgctagtgcatccactaaagttaccgaggatgcccgtgtggcaaaccaagctcaacagctttccaaattctttgagtaatcaaaCTGGAGAAATCAATATGATCGTTTCTGACATCGAAGCTAAAGCCCTCTTag                                                                                                                                                                                                                                                                             >  1:219696‑M2/251‑301 (MQ=255)`  
                                 `ttctaatatgactcactaaaggagacacaccatgttcaaactgattaagaagttaggccaactgctggttcgtatgtacaacgtggaagccaagcgactgaacgatgaggctcgtaaagaggccacacagtcacgcgctctggcgattcgctccaacgaactggctgacagtgcatccactaaagttaccgaggctgcccgtgtggcaaaccaagctcaacagctttccaaattctttgagtaatcaaaCAGGAGAAATCAATATGATCGTTTCTGACATCGAAGCTAACGCCCTCTTaga                                                                                                                                                                                                                                                                            >  1:297901‑M2/250‑301 (MQ=255)`  
                                 `ttctaatacgactcactaaaggagacacaccatgttcaaactgattaagaagttaggccaactgctggttcgtatgtacaacgtggaagccaagcgactgaacgatgaggctcgtaaagaggccacacagtcacgcgctctggcgattcgctccaacgaactggctgacagtgcatccactaaagttaccgaggctgcccgtgtggcaaaccaagctcaacagctttccaaattctttgagtaatcaaaCAGGAGAAATCAATATGATCGTTTCTGACATCGAAGCTAAAGCCCTCTTaga                                                                                                                                                                                                                                                                            >  1:129789‑M2/250‑301 (MQ=255)`  
                                 `ttctaatacgactcactaaaggagacacaccatgttcaaactgattaagaagttaggccaactgctggttcgtatgtacaacgtggaagccaagcgactgaacgatgaggctcgtaaagaggccacacagtcacgagctctggcgattcgctccaacgaactggctgacagtgcatccactaaagttaccgaggctgcccgtgtggcaaaccaagctcaacagctttccaaattctttgagtaatcaaaCAGGAGACATCAATATGATAGTTTCTGACATCGAAGCTAACGCCCGCTTaga                                                                                                                                                                                                                                                                            >  1:363038‑M2/250‑301 (MQ=255)`  
                                  `ttctaatacgactcactaaaggagacacaccatgttcaaactgattaagaatttaggccaactgctggttcgtatgtacaacgtggaagccaagcgactgaacgatgaggctcgtaaagaggccacacagtcacgcgctctggctattcgctccaacgaactggctgacagtgcatccactaaagttaccgaggctgcccgtgtggcaaaccacgctcaacagctttccaaattctttgagtaatcaaTAGGAGAAATCAATATGATCGTTTCTGACCTCGAAGCTAACGCCCTCTTagag                                                                                                                                                                                                                                                                           >  1:36749‑M2/249‑301 (MQ=255)`  
                                  `ttctaatacgactcactaaaggagacacaccatgttcaaactgattaagaagttaggccaactgctggttcgtatgtacaacgtggaagccaagcgactgaacgatgaggctcgtaaagaggccacacagtcacgcgctctggcgattcgctccaacgaactggctgacagtgcatccactaaagttaccgaggctgcccgtgtggcaaaccaagctcaacagctttccaaattctttgagtaatcaaTAGGAGAAATCAATATGATCGTTTCTGACATCGAAGCTAACGCCCTCTTagag                                                                                                                                                                                                                                                                           >  1:442501‑M2/249‑301 (MQ=255)`  
                                  `ttctaatacgactcactaaaggagacacaccatgttcaaactgattaagaagttaggccaactgctggttcgtatgtacaacgtggaagccaagcgactgaacgatgaggctcgtaaagaggccacacagtcacgcgctctggcgattcgctccaacgaactggctgacagtgcatccactaaagttaccgaggctgcccgtgtggcaaaccaagctcaacagctttccaaattctttgagtaatcaaTAGGAGAAATCAATATGATCGTTTCTGACATCGAAGCTAACGCCCTCTTagag                                                                                                                                                                                                                                                                           <  1:560825‑M2/53‑1 (MQ=255)`  
                                  `ttctaatacgactcactaaaggagacacaccatgttcaaactgattaagaagataggccaactgctggttcgtatgtacaacgtggaagccaagcgactgaacgatgaggctcgtaaagaggccacacagtcacgcgctctggcgattcgctccaacgaactggctgacagtgcatccactaaagttaccgaggctgcccgtgtggcaaaccaagctcaacagctttccaaattctttgagtaatcaaTAGGAGAAATCAATATGATCGTTTCTGACATCGAAGCTAACGCCCTCTTagag                                                                                                                                                                                                                                                                           <  1:513622‑M2/53‑1 (MQ=255)`  
                                  `ttctaataagactcactaaaggagaaacaccatgtgcaaactgattaagaagttaggccaactgctggttcgtagatacaacgtggaagccaagcgactgaacgatgaggctcgtaaagaggccacacagacacgcgctctggcgattcgctccaacgaactggctgaaagtgcatccactaaagttacagaggatgcccgtgtggcaaaccaagctcaacagctttccaaattctttgagtaatcaaTAGGAGAAATCAATATGATCGTTTCTGACATCGAGGCTAACGCCCTCTTagag                                                                                                                                                                                                                                                                           <  1:619558‑M2/53‑1 (MQ=255)`  
                                  `tgtataagagacagctaaaggagacacaccatgttcaaactgattaagaagttaggccaactgctggttcgtatgtacaacgtggaagccaagcgactgaacgatgaggctcgtaaagagtccacacagtcacgcgctctggcgattcgctccaacgaactggctgacagtgcatccactaaagttaccgaggctgcccgtgtggcaaaccaagctcaacagctttccaaattctttgagtaatcaaaCAGGAGAAACCATTATGATCGTTTCTGACATCGAAGCTAACGCCCTCTTagag                                                                                                                                                                                                                                                                           <  1:595910‑M2/53‑1 (MQ=255)`  
                                  `tcaaatccgactcaataaaggagacacaccatgttcaaactgattaagaagttaggccaactgctggttcgtatggacaacgtggaagccaagcgactgaacgatgaggctcgtaaagaggccacacagtcacgcgctctggcgattcgctccaacgaactggctgacagtgcatccactaaagttaccgaggctgcccgtgtggcaaaccaagctcaacagctttccaaattctttgagtaatcaaaCAGGAGAAATCAATATGATCGTTTCTGACATCGAAGCTAACGCCCTCTTagag                                                                                                                                                                                                                                                                           <  1:159538‑M2/53‑1 (MQ=255)`  
                                  `aagtaatacgactcactaaaggagacacaccatgttcaaactgattaagaaggtaggccaactgctggttcgtatgtacaacgtggaagccaagcgactgaacgatgaggctcgtaaagaggccacacagtcacgcgctctggcgattcgctccaacgaactggctgacagtgcatccactaaagttaccgaggctgcccgtgtggcaaaccaagctcaacagctttccaaattctttgagtaatcaaTAGGAGAAATCAATATGATCGTTTCTGACATCGAAGCTAACGCCCTCTTagag                                                                                                                                                                                                                                                                           <  1:87297‑M2/53‑1 (MQ=255)`  
                                    `ctaatacgactcactaaaggagacacaccatgttcaaactgattaagaagttaggccaactgctggttcgtatgtacaacgtggaagccaagcgactgaacgatgaggctcgtaaagaggccacacagtcacgcgctctggcgattcgctccaacgaactggctgacagtgcatccactaaagttaccgaggctgcccgtgtggcaaaccaagctcaacagctttccaaattctttgagtaatcaaTAGGAGAAATCAATATGATCGTTTCTGACATCGAAGCTAACGCCCTCTTagagag                                                                                                                                                                                                                                                                         >  1:466165‑M2/247‑301 (MQ=255)`  
                                     `aatccgactcactaaaggagacacaccatgttcaaactgattaagaagttaggccaactgctggttcgtatgtacaacgtggaagccaagcgactgaacgatgaggctcgtaaagaggccacacagtcacgcgctctggcgattcgctccaacgaactggctgacagtgcatccactaaagttaccgaggctgcccgtgtggcaaaccaagctcaacagctttccaaattctttgagtaatcaaaCAGGAGAAATCAATATGATCGTTTCTGACATcgaagctaacgcccctgtctcttat                                                                                                                                                                                                                                                                        >  1:499578‑M2/246‑290 (MQ=255)`  
                                      `aatacgactcactaaaggagacacaccatgttcaaactgattaagaagttaggccaactgctggttcgtatgtacaacgtggaagccaagcgactgaacgatgaggctcgtaaagaggccacacagtcacgcgctctggcgattcgctccaacgaactggctgacagtgcatccactaaagttaccgaggctgcccgtgtggcaaaccaagctcaacagctttccaaattctttgagtaatcaaTAGGAGAAATCAATATGATCGTTTCTGACATCGAAGCTAACGCCCtcttagagctgt                                                                                                                                                                                                                                                                       >  1:93433‑M2/245‑297 (MQ=255)`  
                                              `cactaaaggagacacaccatgttcaaactgattaagaagttaggccaactgctggttcgtatgtacaacgtggaagccaagcgactgaacgatgaggctcgtaaagaggccacacagtcacgcgctctggcgattcgctccaacgaactggctgacagtgcatccactaaagttaccgaggctgcccgtgtggcaaaccaagctcaacagctttccaaattctttgagtaatcaaaCAGGAGAAATCAATATGATCGTTTCTGACATCGAAGCTAACGCCCTCTTAGAGAGCGTCACTAAg                                                                                                                                                                                                                                                               >  1:555543‑M2/237‑301 (MQ=255)`  
                                              `cactaaaggagacacaccatgttcaaactgattaagaagttaggccaactgctggttcgtatgtacaacgtggaagccaagcgactgaacgatgaggctcgtaaagaggccacacagtcacgcgctctggcgattcgctccaacgaactggctgacagtgcatccactaaagttaccgaggctgcccgtgtggcaaaccaagctcaacagctttccaaattctttgagtaatcaaaCAGGAGAAATCAATATGATCGTTTCTGACATCGAAGCTAACGCCCTCTTAGAGAGCGTCACTAAg                                                                                                                                                                                                                                                               >  1:208293‑M2/237‑301 (MQ=255)`  
                                              `cactaaaggagacacaccatgttcaaactgattaagaagttaggccaactgctggttcgtatgtacaacgtggaagccaagcgactgaacgatgaggctcgtaaagaggccacacagtcacgagctctggcgattcgctccaacgaactggctgacagtgcatccactaaagttaccgaggctgcccgtgtggcaaaccaagctcaacagctttccaaattctttgagtaatcaaaCAGGAGAAATCAATATGATCGTTTCTGACATCGAAGCtaacgccctcttagagctgtctcttata                                                                                                                                                                                                                                                               >  1:60849‑M2/237‑289 (MQ=255)`  
                                               `cactaaaggagacacaccatgttcaaactgattaagaagttaggccaactgctggttcgtatgtacaacgtggaagccaagcgactgaacgatgaggctcgtaaagaggccacacagtcacgcgctctggcgattcgctcccacgaactggctgacagtgcatacactaaagttaccgaggctgcccgtgtggcaaaccaagctcaacagctttacaaattctttgagtaatcaaTAGGAGAAATCAATATGATCGTTTCTGACATCGAAGCTAACGCCCTCTTCGAGAGCGCCACTAAGt                                                                                                                                                                                                                                                              >  1:115880‑M2/236‑301 (MQ=255)`  
                                               `cactaaaggagacacaccatgttcaaactgattaagaagttaggccaactgctggttcgtatgtacaacgtggaagccaagcgactgaacgatgaggctcgtaaagaggccacacagtcacgcgctctggcgattcgctccaacgaactggctgacagtgcatccactaaagttaccgaggctgcccgtgtggcaaaccaagctcaacagctttccaaattctttgagtaatcaaTAGGAGAAATCAATATAATCGTTTCTGACATCGAAGctaacgccctcttagagctgtctcttatac                                                                                                                                                                                                                                                              >  1:48263‑M2/236‑288 (MQ=255)`  
                                                 `ctaaaggagacacaccatgttcaaactgattaagaagttaggccaactgctggttcgtatgtacaacgtggaagccaagcgactgaacgatgaggctcgtcaataggccacacagtcacgcgctctggcgattcgctccaacgaactggctgacagtgcatccactaaagttaccgaggctgcccgtgtggcaaaccaagctcaacagctttccaaattctttgagtaatcaaTAGGAGAAATCAATATGATCGTTTCTGACATCGAACCTAACGCCCTCTTAGAGAGCGTCACTAAGTTc                                                                                                                                                                                                                                                            >  1:522993‑M2/234‑301 (MQ=255)`  
                                                 `ctaaaggagacacaccatgttcaaactgattaagaagttaggccaactgctggttcgtatgtacaacgtggaagccaagcgactgaacgatgaggctcgtaaagaggccacacagtcacgcgctctggcgattcgctccaacgaactggctgacagtgcatccactaaagttaccgaggctgcccgtgtggcaaaccaagctcaacagctttccaaattctttgagtaatcaaTAGGAGAAATCAATATGATCGTTTCTGACATCGAAGCTAACGCCCTCTTAGAGAGCGTCACTAAGTTc                                                                                                                                                                                                                                                            <  1:454735‑M2/68‑1 (MQ=255)`  
                                                   `aaaggtgacacaccatgttcaaactgattaagaagttaggccaactgctgattcgtatgtacaacgtggaagccaagcgcctgaacgatgaggctcgtaaacagctcaaacagtaaagcgctcgggcgactcgctccaccgaactgtctgacagtgcagccacgaaagtgaccgagtctgcccgtgtggcaaaccatgctcaaaagctttccaacttctttgagtaatcaaTATGACAAATCAATATGATCGTTTCTGACATCAACGGTAACGCCCTCCTAGAGAGCGTCACtaagtctca                                                                                                                                                                                                                                                          >  1:80898‑M2/232‑297 (MQ=255)`  
                                                      `gagacacaccatgttcaaactgattaagaagttaggccaactgctggttcgtatgtacaacgtggaagccaagcgactgaacgatgaggctcgtaaagaggccacacagtcacgcgctctggcgattcgctccaacgaactggctgacagtgcatccactaaagttaccgaggctgcccgtgtggcaaaccaagctcaacagctttccaaattctttgagtaatcaaaCAGGAGAAATCAATATGATCGTTTCTGACATCGAAGCTAACGCCCTCTTAGAGAGCGTCACTAAGTTCCACTg                                                                                                                                                                                                                                                       >  1:61486‑M2/229‑301 (MQ=255)`  
                                                            `caccatgttcaaactgattaagaagttaggccaactgctggttcgtatgtacaacgtggaagccaagcgactgaacgatgaggctcgtaaagaggccacacagtcacgcgctctggcgattcgctccaacgaactggctgacagtgcatccactaaagttatcgaggctgcccgtgtggcaaaccaagctcaacagctttccaaattctttgagtaatcaaaCAGGAGAAATCAATATGATCGTTTCTGACATCGAAGCTAACGCCCTCTTAGAGAGCGTCACTAAGTTCCACTGCGGGGt                                                                                                                                                                                                                                                 <  1:476826‑M2/79‑1 (MQ=255)`  
                                                             `caccatgttcaaactgattaagaagttaggccaactgctggttcgtatgtacaacgtggaagccaagcgactgaacgatgaggctcgtaaagaggccacacagtcacgcgctctggcgattcgctccaacgaactggctgacagtgcatccactaaagttaccgaggctgcccgtgtggcaaaccaagctcaacagctttccaaattctttgagtaatcaaTAGGAGAAATCAATATGATCGTTTCTGACATCGAAGCTAACGCCCTCTTAGAGAGCGTCACTAAGTTCCACTGCGGGGtt                                                                                                                                                                                                                                                >  1:201906‑M2/222‑301 (MQ=255)`  
                                                             `caccatgttcaaactgattaagaagttaggccaactgctggttcgtatgtacaacgtggaagccaagcgactgaacgatgaggctcgtaaagaggccacacagtcacgcgctctggcgattcgctccaacgaactggctgacagtgcatccactaaagttaccgaggctgcccgtgtggcaaaccaagctcaacagctatacaaattctttgagtaatcaaTAGGAGAAATCAATATGATCGTTTCTGACATCGAAGCTAACGCACTCTTAGAGAGCGTCACTAAGTTCCACTGCGGGGtt                                                                                                                                                                                                                                                >  1:430951‑M2/222‑301 (MQ=255)`  
                                                                 `atgttcaaactgatttagaagttaggccaactgctggttcgtatgtacaacgtggaagccaagcgactgaacgatgaggctcgtaaagaggccacacagtcacgcgctctggcgattcgctcccacgaactggctgacagtgcatccactaaagttaccgaggctgcccgtgtggcaaaccaagctcaacagctttcccaattctttgagtaatcaaTAGGAGAAATCAATATGATCGGTTCTGACATCGAAGCTAACGCCCTCTTAGAGAGCGtcactaagttccacctgtctcttatac                                                                                                                                                                                                                                            >  1:380317‑M2/218‑288 (MQ=255)`  
                                                                   `ttcaaactgattaagaagttaggccaactgctggttcgtatgtacaacgtggaagccaagcgactgaacgatgaggctcgtaaagaggccacacagtcacgcgctctggcgattcgctccaacgaactggctgacagtgcatccactaaagttaccgaggctgcccgtgtggcaaaccaagctcaacagctttccaaattctttgagtaatcaaaCAGGAGAAATCAATATGATCGTTTCTGACATCGAAGCTAACGCCCTCTTAGAGAGCGTCACTAAGTTCCACTGCGGGGTTATCTAc                                                                                                                                                                                                                                          <  1:189773‑M2/86‑1 (MQ=255)`  
                                                                      `caaactgatgaagaagttaggccaactgctggttcgtatgtacaacgtggaagccaagcgactgaacgatgaggctcgtaaagaggccacacagtcacgcgctctggcgattcgctccaacgaactggctgacagtgcatccactaaagttaccgaggctgcccgtgtggcaaaccaagctcaacagctttccaaattctttgagtaatcaaTAGGAGAAATCAATATGATCGTTTCTGACATCGAAGCTAACGCCCTCTTAGAGAGCGTCACTAAGTTCCACTGCGGGGTTATCTACGac                                                                                                                                                                                                                                       <  1:261155‑M2/89‑1 (MQ=255)`  
                                                                            `gattaagaagttaggccaactgctggttcgtatgtacaacgtggaagccaagcgcctgtacgatgaggctcgtaacgaggccacacagtcacgcgctctggcgattagctccaacgaactggctgacagtgcatccactcaagttaccgaggctgcccgtgtggcaaaccaagctcaacagctttccaaattcttttcgtaatcaaTAGTAGAAATCCATATGATCGTTTCTGACATCTAAGCTACCGCCCTCTTAGCGAGCGTCACTAAGTTCCACTGCGGGGTTTTatacgactcttcc                                                                                                                                                                                                                                 >  1:322025‑M2/207‑296 (MQ=255)`  
                                                                             `attaagaagttaggccaactgctggttcgtatgtacaacgtggaagccaagcgactgaacgatgaggctcgtaaagaggccacacagtcacgcgctctggcgattcgctccaacgaactggctgacagtgcatccactaaagttaccgaggctgcccgtgtggcaaaccaagctccacagctttccaaattcttttagtaatcaaTAGGAGAAATCAATATGATCGTTTCTGACATCGAATCTAACGCCCTCTTAGCGATCGTCACTAAGTTCCACTGCGGGGTTATCTacgcctacttcc                                                                                                                                                                                                                                >  1:346028‑M2/206‑298 (MQ=255)`  
                                                                                        `aggccaactgctggttcgtatggacaacggggaagccaagcgacggaacgatgaggctcgtaaagaggccacacagtcacgcgctctggcgattcgctccaacgaactggctgacagtgcatccactaaagttaccgaggctgcccgtgtggcaaaccaagctcaacagctttccaaattctttgagtaatcaaTAGGAGAAATCAATATGATCGTTTCTGACATCGAAGCTAACGCCCTCTTAGAGAGCGTCACTAAGTTCCACTGCGGGGTTATCTACGACTACTCCACCGCTGAGTAc                                                                                                                                                                                                                     <  1:196134‑M2/107‑1 (MQ=255)`  
                                                                                          `gccaactgctggttcgtatgtaaaacgtggaagccaagcgactgaacgatgaggctcgtaaagaggccacacagtcacgcgctctggcgattcgctccaacgaactggctgacagtgcatccactaaagttaccgaggctgcccgtgtggcaaaccaagctcaacagctttccaaattctttgagtaatcaaTAGGAGAAATCAATATGATCGTTTCTGACATCGAAGCTAACGCCCTCTTAGAGAGCGTCACTAAGTTCCACTGCGGGGTTATCTACGACTACTCCACCGCTGAGTACGt                                                                                                                                                                                                                   <  1:225185‑M2/109‑1 (MQ=255)`  
                                                                                             `aactgctggttcgtatgtacaacgtggaagccaagcgactgaacgatgaggctcgtaaagaggccacacagtcacgcgctctggcgattcgctccaacgaactggctgacagtgcatccactaaagttaccgaggctgcccgtgtggcaaaccaagctcaacagctttccaaattctttgagtaatcaaTAGGAGAAATCAATATGATCGTTTCTGACATCGAAGCTAACGCCCTCTTAGAGAGCGTCACTAAGTTCCACTGCGGTGTTATCTACGACTACTCCACCGCTGAGTACGTAAg                                                                                                                                                                                                                <  1:177812‑M2/112‑1 (MQ=255)`  
                                                                                              `ctgctggttcgtatgtacaacgtggaagccaagcgactgaacgatgaggctcgtaaagatgccacacagtcacgcgctctggcgattcgctccaacgaactggctgacagtgcatccactaaagttaccgaggctgcccgtgtggcaaaccaagctcaacagctttccaaattctttgagtaatcaaaCAGGAGAAATCAATATGATCGTTTCTGACATCGAAGCTAACGCCCTCTTAGAGAGCGTCACTAAGTTCCACTGCGGGGTTATCTACGACTACTCCACCGCTGAGTACGTAAGc                                                                                                                                                                                                               >  1:601016‑M2/189‑301 (MQ=255)`  
                                                                                                 `gaagggtcgtatgtacaacatggaagccaagcgactgaacgatgaggctcgtaaagaggccacacagtcacgcgctctggcgattcgctccaacgaactggctgacagtgcatccactaaagttaccgaggctacccgtgtggcaaaccaagctcaacagcttcccaaatgctttgagtaaaaaaTAGGAGAAATCAATATGATCGTTTATGACATCGAAGCTAACGACCTCTTAGAGAGCGTCACTAAGTTCCACTGCGGGGTTATCTACGACTACTCCACCGCTGAGTACGTAAGCTAc                                                                                                                                                                                                            <  1:279828‑M2/116‑1 (MQ=255)`  
                                                                                                         `tatgtacaacgtggaagccaagcgactgaacgatgaggctcgtaaagaggccacacagtcacgcgctctggcgattcgctccaacgaactggctgacagtgcatccactaaagttaccgaggctgcccgtgtggcaaaccaagctcaacagctttccaaattctttgagtaatcaaaCAGGAGAAATCAATATGATCGTTTCTGACATCGAAGCTAACGCCCTCTTAGAGAGCGTCACTAAGTTCCACTGCGGGGTTATCTACGACTACTCCACCGCTGAGTACGTAAGCTACCGTCCGAg                                                                                                                                                                                                    <  1:505650‑M2/124‑1 (MQ=255)`  
                                                                                                            `tgtacaacgtggaagccaagctactgaacgatgatgctcgtaaagaggccacacagtcacgcgctctggcgattcgctccaacgaactggctgacagtgcatccactaaagttaccgaggctgcccgtgtggcaaaccaagctcaacagctttccaaattctttgagtaatcaaTAGGAGAAATCAATATGATCGTTTCTGACATCGAAGCTAACGCCCTCTTAGAGAGCGTCACTAAGTTCCACTGCGGGGTTATCTACGACTACTCCACCGCTGAGTACGTAAGCTACCGTCCGAGTGa                                                                                                                                                                                                 <  1:358931‑M2/127‑1 (MQ=255)`  
                                                                                                             `gtacaacgtggaagccaagcgactgaacgatgaggctcgtaaagcagccacacagtcacgcgcgctggcgattcgctccaacgaactggctgacagtgcatccactaaagttaccgaggctgcccgtgtggcaaaccaagctcaacagctttccaaattatttgagtaatcaaTAGGAGAAATCAATATGATCGTTTCTGACATCGAAGCTAACGCCCTCTGAGAGAGCGTCACTAAGTTCCACTGCGGGGTTATCTACGACTACTCCACCGCTGAGTACGTAAGCTACCGTCCGAGTGAc                                                                                                                                                                                                <  1:27319‑M2/128‑1 (MQ=255)`  
                                                                                                               `acaacgtggaagccaagcgactgaacgatgaggctcgtaaagaggccacacagtcacgcgctctggcgattcgctccaacgaactggctgacagtgcatccactaaagttaccgaggctgcccgtgtggcaaaccaagctcaacagctttccaaattctttgagtaatcaaTAGGAGAAATCAATATGATCGTTTCTGACATCGAAGCTAACGCCCTCTTAGAGAGCGTCACTAAGTTCCACTGCGGGGTTATCTACGACTACTCCACCGCTGAGTACGTAAGCTACCGTCCGAGTGACtt                                                                                                                                                                                              <  1:231308‑M2/130‑1 (MQ=255)`  
                                                                                                               `acaacgtggaagccaagcgactgaacgatgaggctcgtaaagaggccacacagtcacgcgctctggcgattcactacaacgaactggctgacagtgcatccactaaagttacggaggctgcccgtgtggcaaaccaagcgcaacagctttccaaatgctttgagtaatcaaTAGGAGAAATCAATATGATCGTTTCTGATATCGAAGCTAACGCACTCTTAGAGAGCGTCACTAAGTTCCACTGCGGGGTTATCTACGACTACTCCACCGCTGAGTACGTAAGCTACCGTCCGAGTGACtt                                                                                                                                                                                              <  1:541824‑M2/130‑1 (MQ=255)`  
                                                                                                                `aaacgtggaagccaagcgactgaacgatgaggctcgtaaagagtccacacagtcacgcgctatggcgattcgctccaacgaactggctgacagtgcatccactaaagttaccgaggctgcccgtgtggcaaaccaagctcaacagctttccaaattctttgagtaatcaaTAGGAGAAATTAAGATGATCGTTTCTGACATCGAAGCTAACGCCCTCTTAGAGAGCGTCACTAAGTTCCACTGCGGGGTTATCTACGACTACTCCACCGCTGAGTACGTAAGCTACCGTCCGAGTGACTTc                                                                                                                                                                                             <  1:75927‑M2/131‑1 (MQ=255)`  
                                                                                                                          `gccaagcgactgaacgatgaggctcgtaaagaggccacacagtcacgcgctctggcgattcgctccaacgaactggctgacagtgcatccactaaagttaccgaggctgcccgtgtggcaaaccaagctcaacagctttccaaattctttgagtaatcaaTAGGAGAAATCAATATGATCGTTTCTGACATCGAAGCTAACGCCCTCTTAGAGAGCGTCACTAAGTTCCACTGCGGGGTTATCTACGACTACTCCACCGCTGAGTACGTAAGCTACCGTCCGAGTGACTTCGGTGCGTATc                                                                                                                                                                                   <  1:243825‑M2/141‑1 (MQ=255)`  
                                                                                                                              `gcgactgaacgatgaggctcgtaaagaggccacacagtcacgcgctctggcgattcgctccaacgaactggctgacagtgcatccactaaagttaccgaggctgcccgtgtggcaaaccaagctcaacagctttccaaattctttgagtaatcaaaCAGGTGAAATCAATATGATCGTTTCTGACATCGAAGCTAACGCCCTCTTAGAGAGCGTCACTAAGTTCCACTGCGGGGTTATCTACGACTACTCCACCGCTGAGTACGTAAGCTACCGTCCGAGTGACTTCGGTGCGTATCTGGa                                                                                                                                                                               <  1:528883‑M2/145‑1 (MQ=255)`  
                                                                                                                              `gcgactgaacgatgaggctcgtaaagaggccacacagtcacgcgctctggcgattcgctccaacgaactggctgacagtgcatccactaaagttaccgaggctgcccgtgtggcaaaccaagctcaacagctttccaaattctttgagtaatcaaaCAGGAGAAACCATTATGATCGTTTCTGACATCGAAGCTAACGCCCTCTTAGAGAGCGTCACTAAGTTCCACTGCGGGGTTATCTACGACTACTCCACCGCTAAGTACGTAAGCTACCGTCCGAGTGACTTCGGTGCGTATCTGGa                                                                                                                                                                               <  1:582461‑M2/145‑1 (MQ=255)`  
                                                                                                                              `agcgactgaacgatgaggctcgtaaagaggccacacagtcacgcgctctggcgattcgctccaacgaactggctgacagtgcatccactaaagttaccgaggctgcccgtgtggcaaaccaagctcaacagctttccaaattctttgagtaatcaaTAGGAGAAATCAATATGATCGTTTCTGACATCGAAGCTAACGCCCTCTTAGAGAGCGTCACTAAGTTCCACTGCGGGGTTATCTACGACTACTCCACCGCTGAGTACGTAAGCTACCGTCCGAGTGACTTCGGTGCGTATCTGGa                                                                                                                                                                               <  1:249136‑M2/145‑1 (MQ=255)`  
                                                                                                                                    `gaacgatgaggctcgtaaagaggccacacagtcacgcgatctggcgattcgctccaacgaactggctgacagtgcatccactaaagttaccgaggctgcccgtgtggcaaaccaagctcaacagctttccaaattctttgagtaatcaaaCAGGAGAAATCAATATGATCGTTTCTGACATCGAAGCTAACGCCCTCTTAGAGAGCGTCACTAAGTTCCACTGCGGGGTTATCTACGACTACTCCACCGCTGAGTACGTAAGCTACCGTCCGAGTGACTTCGGTGCGTATCTGGATGCGCt                                                                                                                                                                         >  1:13525‑M2/151‑301 (MQ=255)`  
                                                                                                                                     `gaacgatgaggctcgtaaagaggccacacagtcacgcgctctggcgattcgctccaacgaactggctgacagtgcatccactaaagttaccgaggctgcccgtgtggcaaaccaagctcaacagctttccaaattctttgagtaatcaaTAGGAGAAATCAATATGATCGTTTCTGACATCGAAGCTAACGCCCTCTTAGAGAGCGTCACTAAGTTCCACTGCGGGGTTATCTACGACTACTCCACCGCTGAGTACGTAAGCTACCGTCCGAGTGACTTCGGTGCGTATCTGGATGCGCTg                                                                                                                                                                        >  1:287102‑M2/150‑301 (MQ=255)`  
                                                                                                                                                         `aggccacacagtcacgcgctctggcgattcgctcctaagaactggctgacagtgcaaccactaaagttaccgaggctgcccgtgtggcaaaccatgctcaacagctttccaaattctttgagtaatcaaTATGATAAATCAATATGATCGTTTCTGACATCGAAGCTAACGCCCTCTTAGAGAGCGTCACTAAGTTCCACTGCGGGGATATCTACGACTACTCCACCGCTGTGTACGTAAGCTACCGTCCGAATGACTTAGGTGCGTACCTGGATACGCTGGAAGCCGGAGTTGCACGAgg                                                                                                                                                    >  1:105435‑M2/130‑301 (MQ=255)`  
                                                                                                                                                            `ccacacagtcacgcgctctggcgattcgctccaacgaactggctgacagtgcatccactaaagttaccgaggctgcccgtgtggcaaaccaagctcaacagctttccaaattctttgagtaatcaaTAGTAGAAATCAATATGATCGTTTCTGACATCGAAGCTAACGCCCTCTTAGAGAGCGTCACTAAGTTCCACTGCGGGGTTATCTACGACTACTCCACCGCTGAGTACGTAAGCTACCGTCCGAGTGACTTCGGTGCGTATCTGGATGCGCTGGAAGCCGAGGTTGCACGAGGCgg                                                                                                                                                 <  1:236395‑M2/175‑1 (MQ=255)`  
                                                                                                                                                                 `cagtcacgcgctctggcgattcgctccaacgaactggctgacagtgcatccactaaagttaccgatgctgcccgtgtggcaaaccaagctcaacagctttccaaattctttgagtaatcaaTAGGAGAAATCAATATGATCGTTTCTGACATCGAAGCTAACGCCCTCTTAGAGAGCGTCACTAAGTTCCACTGCGGGGTTATCTACGACTACTCCACCGCTGAGTACGTAAGCTACCGTCCGAGTGACTTCGGTGCGTATCTGGATGCGCTGGAAGCCGAGGTTGCACGAGGCGGTcttt                                                                                                                                            >  1:589380‑M2/122‑300 (MQ=255)`  
                                                                                                                                                                   `gtcacgcgctctggcgattcgctccaacgaactggctgacagtgcatccactaaagttaccgaggctgcccgtgtggcaaaccaagctcaacagctttccaaattctttgagtaatcaaTAGGAGAAATCAATATGATCGTTTCTGACATCGAAGCTAACGCCCTCTTAGAGAGCGTCACTAAGTTCCACTGCGGGGTTATCTACGACTACTCCACCGCTGAGTACGTAAGCTACCGTCCGAGTGACTTCGGTGCGTAACTGGATGCGCTGGAAGCCGAGGTTGCACGAGGCGGTCTTAtt                                                                                                                                          >  1:394924‑M2/120‑301 (MQ=255)`  
                                                                                                                                                                    `tcccgcgctctggcaattcgttccaacgaactggctgacagtgcatccactaaagttaccgaggctgcccgtgtggcaaaccaagctcaacagctttccaaattctttgagtaatcaaTAGGAGAAATCAATATGATCGTTTCTGACATCGAAGCTAACGCCCTCTTAGAGAGCGTCACTAAGTTCCACTGCGGGGTTATCTACGACTACTCCACCGCTGAGTACGTAAGCTACCGTCCGAGTGACTTCGGTGCGTATCTGGATGCGCTGGAAGCCGAGGTTGCACGAGGCGGTCTTATtg                                                                                                                                         <  1:641278‑M2/183‑1 (MQ=255)`  
                                                                                                                                                                    `tcacgcgctctgacgattcgctccaacaaactggctgacagtgcatccactaaagttaccgaggctgcccgtgtggcaaaccaagctcaacagctttccaaaaactttgagtaatcaaTAGGAGAAATCAATATGATCGTTTCTGACATCGAAGCTAACGCCCTCTTAGAGAGCGTCACTAAGTTCCACTGCGGGGTTATCTACGACTACTCCACCGCTGAGTACGTAAGCTTCCGTCCGAGTGACTTCGGTGCGTATCTGGATGCGCTGGAAGCCGAGGTTGCACGAGGCGGTCTTATtg                                                                                                                                         <  1:635368‑M2/183‑1 (MQ=255)`  
                                                                                                                                                                           `ctctggcgattcgctccaacgaactggctgacagtgcatccactaaagttaccgaggctgcccgtgtggcaaaccaagctcaacagctttccaaattctttgagtaatcaaTAGGAGAAATCAATATGATCGTTTCTGACATCGAAGCTAACGCCCTCTTAGAGAGCGTCACTAAGTTCCACTGCGGGGTTATCTACGACTACTCCACCGCTGAGTACGTAAGCTACCGTCCGAGTGACTTCGGTGCGTATCTGGATGCGCTGGAAGCCGAGGTTGCACGAGGCGGTCTTATTGTGTtccc                                                                                                                                  >  1:15455‑M2/112‑300 (MQ=255)`  
                                                                                                                                                                            `ctggcgattcgctccaacgaactggctggcagtgcatccactaaagttaccgaggctgcccgtgtggcaaaccaagctcaacagctttccaaattctttgagtaatcaaaCAGGAGAAATCAATATGATCGTTTCTGACATCGAAGCTAACGCCCTCTTAGAGAGCGTCACTAAGTTCCACTGCGGGGTTATCTACGACTACTCCACCGCTGAGTACGTAAGCTACCGTCCGAGTGACTTCGGTGCGTATCTGGATGCGCTGGAAGCCGAGGTTGCACGAGGCGGTCTTATTGTGTTCcac                                                                                                                                 <  1:387224‑M2/191‑1 (MQ=255)`  
                                                                                                                                                                               `ggcgattcgctccaacgaactggctgacagtgcatccactaaagttaccgaggctgcccgtgtggcaaaccaagctcaacagctttccaaattctttgagtaatcaaTAGGAGAAATCAATATGATCGTTTCTGACATCGAAGCTAACGCCCTCTTAGAGAGCGTCACTAATTTCCACTGCGGGGTTATCTACGACTACTCCACCGCTGAGTACGTAAGCTACCGTCCGAGTTACTTCGGTGCGTATCTGGATGCGCTGGAAGCCGAGGTTGCACGAggcggtcttattgctgtcctttat                                                                                                                              >  1:2679‑M2/108‑290 (MQ=255)`  
                                                                                                                                                                                             `ggaactggctgaaagtgcatacactaaagttaccgaggctgcccgtgtggcaaaccaagctcaacagctttccaaagtctttgagtaatcaaaCAGGAGAAATCAATATGATCGTTTCTGACATCGAAGCTAACGCCCTCTTAGAGAGCGTCACTAAGTTCCACTGCGGGGTTATCTACGACTACTCCACCGCTGAGTACGTAAGCTACCGTCCGAGTGACTTCGGTGCGTATCTGGATGCGCTGGAAGCCGAGGTTGCACGAGGCGGTCTTATTGTGTTCCACAACGGTCACAAGTATGa                                                                                                                <  1:310033‑M2/208‑1 (MQ=255)`  
                                                                                                                                                                                             `cgaactggctgacagtgcatacactaaagttaccgaggctgcccgtgtggaaaaccaagctcaacagctttccaaattctttgagtaatcaaaCAGGAGAAATCAATATGATCGTTTCTGACATCGAAGCTAACGCCCTCTTAGAGAGCGTCACTAAGTTCCACTGCGGGGTTATCTACGACTACTCCACCGCTGAGTACGTAAGCTACCGTCCGAGTGACTTCGGTGCGTATCTGGATGCGCTGGAAGCCGAGGTTGCACGAGGCGGTCTTATTGTGTTCCACAACGGTCACAAGTATGa                                                                                                                <  1:400614‑M2/208‑1 (MQ=255)`  
                                                                                                                                                                                                    `ggctgacagtgcatccactaaagttaccgaggctgcccgtgtggcaaaccaagctcaacagctttccaaattctttgagtaatcaaTAGGAGAAATCAATATGATCGTTTCTGACATCGAAGCTAACGCCCTCTTAGAGAGCGTCACTAAGTTCCACTGCGGTGTTATCTACGACTACTCCACCGCTGAGTACGTAATCTACCGTCCGAGTGACTTCGGTGCGTATCTGGATGCGCTGGAAGCCGAGGTTGCACGAGGCGGTCTTATTGTGTTCCACAACGGTCACAAgtatgactgtctt                                                                                                         >  1:184026‑M2/87‑295 (MQ=255)`  
                                                                                                                                                                                                        `gacagtgcatccactaaagttaccgaggctgcccgtgtggcaaaccaagctcaacagctttccaaattctttgagtaatcaaTAGGAGAAATCAATATGATCGTTTCTGACATCGAAGCTAACGCCCTCTTAGAGAGCGTCACTAAGTTCCACTGCGGGGTTATCTACGACTACTCCACCGCTGAGTACGTAAGCTACCGTCCGAGTGACTTCGGTGCGTATCTGGATGCGCTGGAAGCCGAGGTTGCACGAGGCGGTCTTATTGTGTTCCACAACGGTCACAAGTATGACGTTCCTGCAt                                                                                                     <  1:304367‑M2/219‑1 (MQ=255)`  
                                                                                                                                                                                                          `cagtgcatccactaaagttaccgaggctgcccgtgtggcaaaccaagctcaacagctttccaaattctttgagtaatcaaTAGGAGAAATCAATATGATCGTTTCTGACATCGAAGCTCACGCACTCTTAGAGAGCGTCACTAAGTTCCACTGCGGGGTTATGTACGACTACTCCCCCGCTCAGTACGTAAGCTACCGTCCGAGTGACTTCGGTGCGTATCTGGATGCGCTGGAAGCCGAGGTTGCACGAGGCGTTCTTATTGTGTTCCACATCCGTCACAAGTATGACGTTCCTGCattt                                                                                                   >  1:436366‑M2/81‑300 (MQ=255)`  
                                                                                                                                                                                                                        `aagttaccgaggctgcccgtgtggcaaaccaagctcaacagctttccaaattctttgagtaatcaaTAGGAGAAATCAATATGATCGTTTCTGACATCGAAGCTAACGCCCTCTTAGAGAGCGTCACTAAGTTCCACTGCGGGGTTATCTACGACTACTCCACCGCTGAGTACGTAAGCTACCGTCCGAGTGACTTCGGTGCGGATCTGGAGGCGCTGGAAGCCGAGGTTGCACGAGGCGGTCTTATTGGGTTCCACAACGGTCACAAGTATGACGTGCCTGACTTGACAAAACGGGaaaa                                                                                     >  1:315013‑M2/67‑301 (MQ=255)`  
                                                                                                                                                                                                                           `ttaccgaggctgcccgtgtggcaaaccaagctcaacagctttccaaattctttgagtaatcaaTAGGAGAAATCAATATGATCGTTTCTGACATCGAAGCTAACGCCCTCTTAGAGAGCGCCACTAAGTTCCACTGCGGGGTTATCTACGACTACTCCACCGCTGAGTACGTAAGCTACCGTCCGAGTGACTTCGGTGCGTATCTGTATGCGCTGGAAGCAGAGGTTGCACGAGGCGGTCTTCTTGTGTTCcacaacggttacaagtatgtcgttccccacttgccccacctgtcaaagat                                                                                  >  1:648576‑M2/64‑277 (MQ=255)`  
                                                                                                                                                                                                                            `taccgaggctgcccgtgtggcaaaccaagctcaacagctttccaaattctttgagtaatcaaTAGGAGAAATCAATATGATCGTTTCTGACATCGAAGCTAACGCCCTCTTAGAGAGCGTCACTAAGTTCCACTGCGGGGTTATCTACGACTACTCCACCGCTGAGTACGTAAGCTACCGTCCGAGTGACTTCGGTGCGTATCTGGATGCGCTGGAAGCCGAGGTTGCACGAGGCGGTCTTATTGTGTTCCACAACGGTCACAAGTATGACGTTCCTGCATTGACCAAACTGGCAAAGTTg                                                                                 >  1:339786‑M2/63‑301 (MQ=255)`  
                                                                                                                                                                                                                                     `gcccgtgtggcaaaccaagctcaacagctttccaaattctttgagtaatcaaaCAGGAGAAATCAATATGATCGTTTCTGACATCGAAGCTAACGCCCTCTTAGAGAGCGTCACTAAGTTCCACTGCGGGGTTATCTACGACTACTCCACCGCTGAGTACGTAAGCTACCGTCCGAGTGACTTCGGTGCGTATCTGGATGCGCTGGAAGCCGAGGTTGCACGAGGCGGTCTTATTGTGTTCCACAACGGTCACAAGTATGACGTTCCTGCATTGACCAAACTGGCAAAGTTGCAATTGAAc                                                                        <  1:352575‑M2/248‑1 (MQ=255)`  
                                                                                                                                                                                                                                                 `aaccaagctcaacagctttccaaatgctttgagtaatcaaaCAGGAGAAATCAATATGATCGTTTCTGACATCGAAGCTAACGCCCTCTTAGAGAGCGTCACTAAGTTCAACTGCGGGGTTATCTACGACTACTCCACCGCTGAGTACGTAAGCTACCGTCCGAGTGACTTCGGTGCGTATCTGGATGCGCTGGAAGCCGAGGTTGCACGAGGCGGTCTTATTGTGTTCCACAACGGTCACAAGTATGACGTTCCTGCGTTGACCAAACTGGCAAAGTTGCAATTGAACCGAGAGTTCCAc                                                            <  1:255215‑M2/260‑1 (MQ=255)`  
                                                                                                                                                                                                                                                 `aaaccaagctcaacagcgttccaaattctttgagtaatcaaTAGGAGAAATCAATATGATCGTTTCTGACATCGAAGCTAACGCCCTCTTAGAGAGCGTCACTAAGTTCCACTGCGGGGTTATCTACGACTACTCCACCGCTGAGTACGTAAGCTACAGTCCGAGTGACTTCGGTGCGTATCTGGATGCGCTGGAAGCCGAGGTTGCACGAGGCGGTCTTATTGTGTTCCACAACGGTCACAAGTATGACGTTCCTGCATTGACCAAACTGGCAAAGTTGCAATTGAACCGAGAGTTCCAc                                                            <  1:412032‑M2/260‑1 (MQ=255)`  
                                                                                                                                                                                                                                                   `accaagctcaacagctttccaaattctttgagtaatcaaTAGGAGAAATCAATATGATCGTTTCTGACATCGAAGCTAACGCCCTCTTAGAGAGCGTCACTAAGTTCCACTGCGGGGTTATCTACGACTACTCCACCGCTGAGTACGTAAGCTACCGTCCGCGTGACTTCGGTGCTTTTCTGGATGCGCTGGAAGCCGAGGTTGCACGAGGCGTTCTTATTGTGTTCCACACCGGTCACCATTATGACGTTCCTGCATTGACCAAACTGGCAAAGTTGCCATTAAACCCAACGTTCCACCt                                                          >  1:164544‑M2/40‑301 (MQ=255)`  
                                                                                                                                                                                                                                                        `ctcaacagctttccaaattctttgagtaatcaaaCAGGAGAAATCAATATGATCGTTTCTGACATCGAAGCTAACGCCCTCTTAGAGAGCGTCACTAAGTTCCACTGCGGGGTTATCTACGACTACTCCACCGCTGAGTACGTAAGCTACCGTCCGAGTGACTTCGGTGCGTATCTGGATGCGCTGGAAGCCGAGGTTGCACGAGGCGGTCTTATTGTGTTCCACAACGGTCACAAGTATGACGTTCCTGCATTGACCAAACTGGCAAAGTTGCAATTGAACCGAGAGTTCCACCTTCCTc                                                     >  1:534506‑M2/35‑301 (MQ=255)`  
                                                                                                                                                                                                                                                         `ctcaacagctttccaaattctttgagtaatcaaTAGGAGAAATCAATATGATCGTTTCTGACATCGAAGCTAACGCCCTCTTAGAGAGCGTCACTAAGTTCCACTGCGGGGTTATCTACGACTACTCCACCGCTGAGTACGTAAGCTACCGTCCGAGTGACTTCGGTGCGTATCTGGATGCGCTGGAAGCCGAGGTTGCACGAGGCGGTCTTATTGTGTTCCACAACGGTCACAAGTATGACGTTCCTGCATTGACCAAACTGGCAAAGTTGCAATTGAACCGAGAGTTCCACCTTCCTCg                                                    <  1:568081‑M2/268‑1 (MQ=255)`  
                                                                                                                                                                                                                                                                `ggggctcggagatgtgtataagagacagggAGAAATCAATATGATCGTTTCTGACATCGAAGCTAACGCCCTCTTAGAGAGCGTCACTAAGTTCCACTGCGGGGTGATCTACGACTACTCCACCGCTGAGTACGTAAGCTACCGTCCGAGTGACTTCGGTGCGTATCTGGATGCGCTGGAAGCCGAGGTTGCACGAGGCGGTCTTATTGTGTTCCACAACGGTCACAAGTATGACGTTCCTGCATTGACCAAACTGGCAAAGTTGCAATTGAACCGAGAGTTCCACCTTCCTCGTGAGAAc                                             <  1:489137/273‑1 (MQ=255)`  
                                                                                                                                                                                                                                                                `gatgtgtataagagacaggtaatcaaTAGGAGAACTCAATATGATCGTTTCTGACATCGAAGCTAACGCCCTCTTAGAGAGCGTCACTAAGTTCCACTGCGGGGTTATCTACGACTACTCCACCGCTGAGTACGTAAGCTACCGTCTGAGTGACTTCGGTGCGTATCTGGATGCGCTGGAAGCCGAGGTTGCACGAGGCGGTCTTATTGTGTTGCACAACGGTCACAAGTATGACGTTCCGGAATTGACCAAACTGGCAAAGTTGCAAGGGAACCGAGAGTTCCACCGTCCTCGTGAGAAc                                             <  1:110518‑M2/275‑1 (MQ=255)`  
                                                                                                                                                                                                                                                                   `tccaaattctctgagtaatcaaaCAGGAGAAATCAATATGATCGTTTCTGACATCGAAGCTAACGCCCTCTTAGAGAGCGTCACTAAGTTCCACTGCGGGGTTATCTACGACTACTCCACCGCTGAGTACGTAAGCTACCGTCCGAGTGACTTCGGTGCGTATCTGGATGCGCTTGAAGCCGAGGTTGCACGAGGCGGTCTTATTGTGTTCCACAACGGTCACAAGTATGACGTTACTGCATTGACCAAACTGGCCAAGTTGCAATTGAACCGAGCGTTCCACCTTCCTCGTGAGAACTGt                                          >  1:327400‑M2/24‑301 (MQ=255)`  
                                                                                                                                                                                                                                                                        `aattctttgagtaatcagTAGGAGAAATCAATATGATCGGTCATGGCATCGAACCTAACGACATCGTAAAGAGCGTCACTAAGTTCCACTGCTGGGTTATCTACGACTAATCCACCGCTGAATACGTAAGCTAGCGTCCGAGTGACTTCGGTGCGTATCTGGATGCGCTAGATGCCGAGGTTGCACGAGGCGGTCTTATTGTGTTCCACAACGTTCACAAGTATGACGTTCATGCATTGACCAAACTGGCAAAGTTGCAATTGAACCGAGAGTTCCACCTTCCTCGTGAGAACTGTATTGa                                     <  1:625007‑M2/283‑1 (MQ=255)`  
                                                                                                                                                                                                                                                                         `tggaaaagagaaagaaaaagtagaAATCCATATGATCGTTTCTGACAGCGAAGCTACCGCCCTCTTAGAGAGCGTCACTAAGTTCCACTGCGGGGTTATCTACGACTACTCCACCGCTGAGTACGTAAGCTACCGTCCGAGTGACTTCGGTGCGTATCTGGATGCGCTGGAAGCCGAGGTTGCACGAGGCGGTCTTATTGTGCTCCACAACGGTCACAAGTATGACGTTCCTGCATTGACCAAACTGGCAAAGTTGCAATTGAACCGAGAGTTCCACCTTCCTCGTGAGAACTGTATTGac                                    <  1:630553/280‑1 (MQ=255)`  
                                                                                                                                                                                                                                                                         `tataagagacagatcaaTAGGAGAAATCAATATGATCGTTTCTGACATCGAAGCTAACGCCCTCTTAGAGAGCGTCACTAAGGTCCACTGCGGGGTTATCTACGACTACTCCACCGCTGAGTACGTAAGCTACCGTCCGAGTGACTTCGGTGCGTATCTGGATGCGCTGGAAGCCGAGGTTGCACGAGGCGGTCTTATTGTGTTCCACAACGGTCACAAGTATGACGTTCCTGCATTGACCAAACTGGCAAAGTTGCAATTGAACCGAGAGTTCCACCTTCCTCGTGAGAACTGTATTGac                                    <  1:575940‑M2/284‑1 (MQ=255)`  
                                                                                                                                                                                                                                                                         `attctttgagtaatcaaTAGGAGAAATCAATATGATCGTTTCTGACATCGAAGCTAACGCCCTCTTAGAGAGCGTCACTAAGTTCCACTGCGGGGTTATCTACGACTACTCCACCGCTGAGTACGTAAGCTACCGTCCGAGTGACTTCGGTGCGTATCTGGATGCGCTGGAAGCCGAGGTTGCACGAGGCGGTCTTATTGTGTTCCACAACGGTCACAAGTATGACGTTCCTGCATTGACCAAACTGGCAAAGTTGCAATTGAACCGAGAGTTCCACCTTCCTCGTGAGAACTGTATTGac                                    <  1:667196‑M2/284‑1 (MQ=255)`  
                                                                                                                                                                                                                                                                         `attctttgagtaataaaTAGGAGAAATCAATATGATCGTTTCTGACATCGAAGCTAACGCCCTCTTAGAGAGCGTCACTAAGTTCCACTGCGGGGTTATCTACGACTACTCCACCGCTGAGTATGTAAGCTACCGTCCGAGTGACTTCGGTGCGTATCTGGATGCGCTGGAAGCCGAGGTTGCACGAGGCGGTCTTATTGTGTTCCACAACGGTCACAAGTATGACGTTCCTGCATTGACCAAACTGGCAAAGTTGCAATTGAACCGAGAGTTCCACCTTCCTCGTGAGAACTGTATTGac                                    <  1:5497‑M2/284‑1 (MQ=255)`  
                                                                                                                                                                                                                                                                                `gagtaatcaaTAGGAGAAATCAATATGATCGTTTCTGACATCGAAGCTAACGCCCTCTTAGAGAGCGTCACTAAGTTCCACTGCGGGGTTATCTACGACTACTCCACCGCTGAGTACGTAAGCTACCGTCCGAGTGACTTCGGTGCGTATCTGGATGCGCTGGAAGCCGAGGTTGCACGAGGCGGTCTTATTGTGTTCCACAACGGTCACACGTATGACGTTCCTGCATTGACCAAACTGGCAAAGTTGCAATTGAACCGAGAGTTCCACCTTCCTCGTGAGAAATGTATTgacacctgtc                             >  1:142292‑M2/11‑297 (MQ=255)`  
                                                                                                                                                                                                                                                                                  `cagtcaaacaGGAGAAATCAATATGATCGTTTCTGACATCGAAGCTAACGCCCTCTTAGAGAGCGTCACTAAGTTCCACTGCGGGGTTATCTACGACTACTCCACCGCTGAGTACGTAAGCTACCGTCCGAGTGACTTCGGTGCGTATCTGGATGCGCTGGAAGCCGAGGTTGCACGAGGCGGTCTTATTGTGTTCCACAACGGTCACAAGTATGACGTTCCTGCATTGACCAAACTGGCAAAGTTGCAATTGAACCGAGAGTTCCACCTTCCTCGTGAGAACTGTATTGACACCCTtgtg                           <  1:493845/292‑1 (MQ=255)`  
                                                                                                                                                                                                                                                                                   `aaagcaaTAGGAGAAATCAATAGGAGCGTTGCTGACATCGAAGCTAACGACCTCTTAGAGAGCGTCACTAAGTTCAACTGCGGGTTTATCTACGAATAATCAACCGCGGAGTACGTAAGCTACAGTCCGAGTGACTTCGGTGCGTATCTGGATGCGCTGGAAGCCGAGGTTGCACGAGGCGGTCTTATTGTGTTCCACAACGGTCACAAGTATGACGTTCCTGCATTGATCAAACTGGCAAAGTTGCAATTGAACCGAGAGTTCCACCTTCCTCGTGAGAACTGTATTGACACCCTTgtgt                          <  1:660541‑M2/294‑1 (MQ=255)`  
                                                                                                                                                                                                                                                                                     `atcaaTAGGAGAAATCAATATGATCGTTTCTGACATCGAAGCTAACGCCCTCTTAGAGAGCGTCACTAAGTTCCACTGCGGGGTTATCTACGACTACTCCACCGCTGAGTACGTAAGCTACCGTCCGAGTGACTTCGGTGCGTATCTGGCTGCGCTGGTAGCCGAGGTTGCACGAGGCGGCCTTATTGTGTTCCACAACGGTCACAAGTATGACGTTCCTGCATTGACCAAACTGGCAAAGTTGCAATTGAACCGAGAGTTCCACCTTCCTAGTGAGAACTGTATTGACACCCTTgtgttt                        >  1:420087‑M2/6‑300 (MQ=255)`  
                                                                                                                                                                                                                                                                                       `aaacaGGAGAAATCAATATGATCGTTTCTGCCATCGAAGCTAACGCCCTCTTAGAGAGCGTCACTAAGTTCCACTGCGGGGTTATCTACGACTACTCCACCGCTGAGTACGTAAGCTACCGTCCGAGTGACTTCGGTGCGTATCTGGATGCGCTGGAAGCCGAGGTTGCACGAGGCGGTCTTATTGTGTTCCACAACGGTCACAAGTATGACGTTCCTGCATTGACCAAACTGGCAAAGTTGCAATTGAACCGAGAGTTCCACCTTCCTCGTGAGCACTGTATTGACACCCTTGtgttgtt                      >  1:122972/5‑300 (MQ=255)`  
                                                                                                                                                                                                                                                                                            `ggAGAAATCAATATGATCGTTTCTGACATCGAAGCTAACGCCCTCTTAGAGAGCGTCACTAAGTTCCACTGCGGGGTTATCTACGACTACTCCACCGCTGAGTACGTAAGCTACCGTCCGAGTGACTTCGGTGCGTATCTGGATGCGCTGGAAGCCGAGGTTGCACGAGGCGGTCTTATTGTGTTCCCCAACGGTCACAAGTATGACGTTCCTGCATTGACCAAACTGGCAAAGTTGCAATTGAACCGAGAGTTCCACCTTCCTCGTGAGAACTGTATTGACACCCTTGTGTTGTCACGtt                 >  1:472204/1‑301 (MQ=255)`  
                                                                                                                                                                                                                                                                                            `ggAGAAATCAATATGATCGTTTCTGACATCGAAGCTAACGCCCTCTTAGAGAGCGTCACTAAGTTCCACTGCGGGGTTATCTACGACTACTCCACCGCTGAGTACGTAAGCTACCGTCCGAGTGACTTCGGTGCGTATCTGGATGCGCTGGAAGCCGAGGTTGCACGAGGCGGTCTTATTGTGTTCCACAACGGTCACAAGTATGACGTTCCTGCATTGACCAAACTGGCAAAGTTGCAATTGAACCGAGAGTTCCACCTTCCTCGTGAGAACTGTATTGACACCCTTgtgtctgtctctt                 >  1:374843/1‑292 (MQ=255)`  
                                                                                                                                                                                                                                                                                                 `aaTCAATATGATCGTTTCTGACATCGAAGCTAACGCCCTCTTAGAGAGCGTCACTAAGTTCCACTGCGGGGTTATCTACGACTACTCCACCGCTGAGTACGTAAGCTACCGTCCGAGTGACTTCGGTGCGTATCTGGATGCGCTGGAAGCCGAGGTTGCACGAGGCGGTCTTATTGTGTTCCACAACGGTCACAAGTATGACGTTCCTGCATTTACCAAACTGGCAAAGTTGCAATTGAACCGAGAGTTCCACCTTCCTCGTGAGAACTGTATTGactgtctcttatacacatctccgagc            >  1:519065/1‑277 (MQ=255)`  
                                                                                                                                                                                                                                                                                                  `aTCAATATGATCGTGTCTGACATCGAAGCTAACGCCCTCTTAGAGAGCGTCACTAAGTTCCACTGCGGGGTTATCTACGACTACTCCACCGCTTAGTACGTAAGCTACCGTACGAGTGACTTCGGTGCGTATCTGGATGCGCTGGAAGCCGAGGTTGCACGAGGCGGTCTTATTGTGTTCCACAACGGTCACAAGTATGACGTTCCTGCATTGACCAAACTGGCAAAGTTGCAATTGAACCGAGAGTTCCACCTTCCTCGTGAGAACTGTATTGACACCCTTGTGTTGTCACGTTTGattc           <  1:45887/301‑1 (MQ=255)`  
                                                                                                                                                                                                                                                                                                    `cAATATGATCGTTTCTGACATCGAAGCTAACGCCCTCTTAGAGAGCGTCACTAAGTTCCACTGCGGGGTTATCTACGACTACTCCACCGCTGAGTACGTAAGCTACCGTCCGAGTGACTTCGGTGCGTATCTGGATGCGCTGGAAGCCGAGGTTGCACGAGGCGGTCTTATTGTGTTCCACAACGGTCACAAGTATGACGTTCCTGCATTGACCAAACTGGCAAAGTTGCAATTGAACCGAGAGTTCCACCTTCCTCGTGAGAACTGTATTGACACCCTTgtgtctgtctcttatacacat         >  1:171870/1‑284 (MQ=255)`  
                                                                                                                                                                                                                                                                                                    `cAATATGATCGTTTCTGACATCGAAGCTAACGCCCTCTTAGAGAGCGTCACTAAGTTCCACTGCGGGGTTATCTACGACTACTCCACCGCTGAGTACGTAAGCTACCGTCCGAGTGACTTCGGTGCGTATCTGGATGCGCTGGAAGCCGAGGTTGCACGAGGCGGTCTTATTGTGTTCCACAACGGTCACAAGTATGACGTTCCTGCATTGACCAAACTGGCAAAGTTGCAATTGAACCGAGAGTTCCACCTTCCTCGTGAGAACTGTATTGACACCCTTGTGTTGTCACGTTTGattcat         >  1:292868/1‑301 (MQ=255)`  
                                                                                                                                                                                                                                                                                                      `atatGATCGTTTCTGACATCGAAGCTAACGCCCTCTTAGAGAGCGTCACTAAGTTCCACTGCGGGGTTATCTACGACTACTCCACCGCTGAGTACGTAAGCTACCGTCCGAGTGACTTCGGTGCGTATCTGGATGCGCTGGAAGCCGAGGTTGCACGAGGCGGTCTTATTGTGTTCCACAACGGTCACAAGTATGACGTTCCTGCATTGACCAAACTGGCAAAGTTGCAATTGAACCGAGAGTTCCACCTTCCTCGTGAGAACTGTATTGACACCCTTGTGTTGTCACGTTTGattcattc       >  1:333947/1‑301 (MQ=255)`  

                                                                                                                                                                                                                                                                                                           `|`                                                                                                                                                                                                                                                                                                              
`GACAGACTGGCTGGAAGAAACCAGAAGTTACCAAGCTGAGTCATAAGGTGTTCGATAAGGACACTATGACCCACATCAAGGCTGGTGATTGGGTTAAGGTTGACATGGGAGTTGTTGGTGGATACGGCTACGTCCGCTCAGTTAGTGGCAAATATGCACAAGTGTCATACATCACAGGTGTTACTCCACGCGGTGCAATCGTTGCCGATAAGACCAACATGATTCACACAGGTTTCTTGACAGTTGTTTCATATGAAGAGATTGTTAAGTCACGATAATCAATAGGAGAAATCAATATGATCGTTTCTGACATCGAAGCTAACGCCCTCTTAGAGAGCGTCACTAAGTTCCACTGCGGGGTTATCTACGACTACTCCACCGCTGAGTACGTAAGCTACCGTCCGAGTGACTTCGGTGCGTATCTGGATGCGCTGGAAGCCGAGGTTGCACGAGGCGGTCTTATTGTGTTCCACAACGGTCACAAGTATGACGTTCCTGCATTGACCAAACTGGCAAAGTTGCAATTGAACCGAGAGTTCCACCTTCCTCGTGAGAACTGTATTGACACCCTTGTGTTGTCACGTTTGATTCATTCCAACC  >  NC_001604.1/14057‑14656`

  

Alignment Legend

Aligned base mismatch/match (shaded by quality score): `ATCG/ATCG` `< 3 ≤` `ATCG/ATCG` `< 10 ≤` `ATCG/ATCG` `< 21 ≤` `ATCG/ATCG` `< 34 ≤` `ATCG/ATCG` `< 38 ≤` `ATCG/ATCG`

Unaligned base: `atcg`    Masked matching base: `atcg`    Alignment gap: `‑`    Deleted base: `‑`

Reads not counted as support for junction

read\_name Not counted due to insufficient overlap past the breakpoint.

read\_name Not counted due to not crossing MOB target site duplication.
